# Activation and tolerance of *Siegesbeckia Orientalis* L. rhizosphere to Cd stress

**DOI:** 10.3389/fpls.2023.1145012

**Published:** 2023-03-24

**Authors:** Jianyu Xie, Xiaoxun Xu, Shirong Zhang, Zhanbiao Yang, Guiyin Wang, Ting Li, Yulin Pu, Wei Zhou, Changlian Xu, Guochun Lv, Zhang Cheng, Junren Xian, Zhien Pu

**Affiliations:** ^1^College of Environmental Sciences, Sichuan Agricultural University, Chengdu, China; ^2^Key Laboratory of Soil Environment Protection of Sichuan Province, Chengdu, China; ^3^College of Resources, Sichuan Agricultural University, Chengdu, China; ^4^College of Agronomy, Sichuan Agricultural University, Chengdu, China

**Keywords:** Cd, rhizosphere, bioavailability, *Siegesbeckia orientalis*, phytoremediation, hyperaccumulator, phytoremediation

## Abstract

This experiment investigated the changes of rhizosphere soil microenvironment for hyperaccumulation-soil system under Cd stress in order to reveal the mechanism of hyperaccumulation and tolerance. Thus, Cd fractions, chemical compositions, and biochemical characteristics in rhizosphere soil of *Siegesbeckia orientalis* L. under Cd stress conditions of 0, 5, 10, 25, 50, 100, and 150 mg kg-1 were investigated through a root bag experiment, respectively. As a result, Cd induced the acidification of *S. orientalis* rhizosphere soil, and promoted the accumulation of dissolved organic carbon (DOC) and readily oxidizable organic carbon (ROC), which increased by 28.39% and 6.98% at the maximum compared with control. The percentage of labile Cd (acid-soluble and reducible Cd) in soil solution increased significantly (P < 0.05) from 31.87% to 64.60% and from 26.00% to 34.49%, respectively. In addition, rhizosphere microenvironment can alleviate the inhibition of Cd on soil microorganisms and enzymes compare with bulk soils. Under medium and low concentrations of Cd, the rhizosphere soil microbial biomass carbon (MBC), basal respiration, ammonification and nitrification were significantly increased (P < 0.05), and the activities of key enzymes were not significantly inhibited. This suggests that pH reduction and organic carbon (DOC and ROC) accumulation increase the bioavailability of Cd and may have contributed to Cd accumulation in *S. orientalis*. Moreover, microorganisms and enzymes in rhizosphere soils can enhance *S. orientalis* tolerance to Cd, alleviating the nutrient imbalance and toxicity caused by Cd pollution. This study revealed the changes of physicochemical and biochemical properties of rhizosphere soil under Cd stress. Rhizosphere soil acidification and organic carbon accumulation are key factors promoting Cd activation, and microorganisms and enzymes are the responses of Cd tolerance.

## 1 Introduction

Cadmium (Cd) is one of the primary heavy metal (HM) pollutants in soils due to its toxicity and non-biodegradability (Lu et al., 2015; Niu et al., 2020). The concentration of Cd in arable soil in China is seriously disturbed by human. Regionally, the maximum Cd concentrations in arable soil around mining and smelting activities, irrigation area by wastewater, urban and suburban area, and remote areas was 152.95, 54.05, 3.15, 2.04 mg kg-1 respectively (Zhang et al., 2015b). Cd in the soil can be easily absorbed by plant roots and then enters the food chain, causing a threat to living creatures and human beings *via* biomagnification and amplification (Salt and Wagner, 1993; Xin et al., 2015). In the past decades, significant progress has been made in the remediation techniques on Cd-contaminated soils (Liu et al., 2021). Among them, phytoextraction technology based on HMs accumulators or hyperaccumulators has been widely used to remediate contaminated soils due to its cost-effective and eco-friendly characteristics (Huang et al., 2018; Moreira et al., 2019). These accumulators can effectively mobilize metals mainly by changing the bioavailability of HMs surrounding roots, enhancing the uptake, and translocation of HMs in plants (Bolan et al., 2011; Zhan et al., 2018). Therefore, the root-soil system is considered to be the key process for phytoextraction, and is increasingly attracting the attention of scholars (Antoniadis et al., 2017; Zhan et al., 2018; Li Y. et al., 2021).

The rhizosphere effect and soil chemical processes that are located within the root-soil interface directly affect the uptakes/exclusions of HMs by plants (Antoniadis et al., 2017). Researches have shown that the chemical conditions of rhizosphere soil were different from those of bulk soil. These chemical conditions can lead to variations of soil compositions, and bioavailability changes of HMs in soil (Chaignon et al., 2002). Solubility and bioavailability of trace metals in the root region and microbial exudates can be adjusted by dissolved organic carbon (DOC) and pH by Rhizosphere effect (Shen et al., 2017; De Conti et al., 2018). Rehman et al. (2017) and Yang et al. (2017) have proved that the decrease of soil pH through rhizosphere effect is the most important single process affecting the availability of HMs. Cd ions absorbed by soil colloids can be exchanged by H^+^ leading to the increase of concentration of Cd^2+^ in root-soil system. In addition, some researches have indicated that rhizosphere effect is induced by metal complexations of DOC. However, rhizosphere effect is very complicated because different results can be obtained even though the conditions are the same. Therefore, it is necessary to further elucidate the mechanisms for activating soil HMs in different plants.

The decrease of pH causes more H+ to exchange with Cd adsorbed by soil colloid and increase the content of Cd2+ in root-soil system, thus increasing Cd mobility (Li Y, et al., 2021). It has been reported that the pH of hyperaccumulators grown in Cd contaminated soil has a lower rhizosphere soil pH than that grown in uncontaminated soil, which increases the uptake of Cd by plants (Gonzaga et al., 2009; Li et al., 2011). Acidification may be ascribed to the secretion of some organic matters including DOC induced by hyperaccumulators (Rehman et al., 2017). DOC, which DOC is a part of soil activated carbon component, which includes low molecular weight organic acids that activate HMs in soil by forming soluble complexes with them (Luo et al., 2017). Soil active organic carbon components are composed of oxidized carbon (ROC) and water-soluble organic carbon (WSOC). ROC is a very abundant active organic carbon component, including easily decomposed humus and polysaccharides (Jia et al., 2015). Humus contains a large number of carboxyl and phenolic hydroxyl acid groups (Bai et al., 2018). These functional groups can change the solution and sorption equilibrium of HMs in soil, and react with metal ions in soil forming soluble complexes. This phenomenon promotes the absorption of metal elements by plants (Ondrasek et al., 2018). WSOC has positive effects on soil microorganisms under HMs stress (Epelde et al., 2010), but there are few studies on the relationship between WSOC and HMs activation. Although rhizosphere acidification and increase of DOC content play an important role in HM activation and accumulations in plants (Lux et al., 2011; Li et al., 2014; Xin et al., 2017). However, how rhizosphere processes help hyperaccumulators tolerance and accumulation of HMs have not been fully explained, and whether ROC and WSOC affect the activation of HMs in rhizosphere is rarely reported.

Soil biochemical properties are ecologically relevant indicators of soil quality, and are often used to evaluate the ecological status of soil under HMs stress (Yang et al., 2017). The researches indicate that microorganisms and enzymes in Pakchoj, black locust, and other nonaccumulators are inhibited by Cd contaminations (Shentu et al., 2014; Xian et al., 2015; Huang et al., 2016; Zhou et al., 2017). However, a completely different phenomenon is now observed in hyperaccumulators. Liu et al. (2020) found that the microorganisms counts and microbial metabolic activity of the hyperaccumulator *Trifolium repens* increased with the increase of Cd supply; a similar phenomenon was also found in Yang’s work (Yang et al., 2017; Niu et al., 2020). Liu et al. (2020) suggested that the relative abundance of plant growth promoting bacteria (*Kaistobacter* and *Flavisolibacter*) and the utilization of difficultly metabolized compounds in rhizosphere would increase under HM stress, which may help alleviate the damage of heavy metals on hyperaccumulators. Yang et al. (2017) found that the remediation process using *S. alfredii* favored Gram-negative bacteria growth more than the Gram-positive bacteria. Niu et al. (2020) under HM stress, rhizosphere soil CAT activity increased in Indian mustard and tall fescue, which accelerated the release of hydrogen peroxide and led to the increase of bacterial 16S rRNA abundance. Therefore, it is necessary to further study which microbial composition and enzyme activity indicators play a key role in the newly discovered hyperaccumulators or different species.

*Siegesbeckia orientalis* L. is promised as an ideal material plant for *in situ* restoring Cd contaminated soils due to its high biomass and Cd concentrating ability in the aboveground (Zhang et al., 2013). Although previous works have investigated roots actively responded to Cd stress and the regulatory mechanisms of HM detoxification(Xu et al., 2020), how to explain that Cd is activated by *S. orientalis* from the root-soil interface and rhizosphere microenvironment is tolerated by Cd stress is still a challenge.

This study hypothesized that HMs were activated by rhizosphere effect originated from change of soil physicochemical properties, while the increase of rhizosphere soil microbial activity and enzyme activity is to enhance *S. orientalis* tolerance to Cd. Therefore, it was assumed that the physicochemical properties in rhizosphere soil will change under the stress of Cd, thus affecting the effectiveness of Cd and the absorption of Cd by *S. orientalis*. Enzymatic activity and microbial activity are also enhanced to maintain normal plant physiological functions. Therefore, the aim of this study was to (1) assess the capacity of *S. orientalis* to uptake Cd from soil; (2) investigate the effects of physicochemical properties rhizosphere of soil on Cd activation; (3) determine the effects of Cd stress on enzyme activities and microbial characteristics of rhizosphere soil.

## 2 Material and methods

### 2.1 Experiment design

*S. orientalis* seeds were harvested from a Pb-Zn mine area in South-West of Chengdu, Sichuan, China (102°46′E, 26°40′N). Seeds were pre-germinated at a plate and kept moist for further treatment. After germination, the seedlings were placed in sandy soil uncontaminated with heavy metal and watered with 1/2 Hoagland solution. After 4 weeks of sand culture, seedling of equal height, health, and leaf number (5 to 6 leaves and a height of about 6 cm) were selected for further treatment study in Cd stressed soil and control study (Xu et al., 2018).

**Figure 1:**
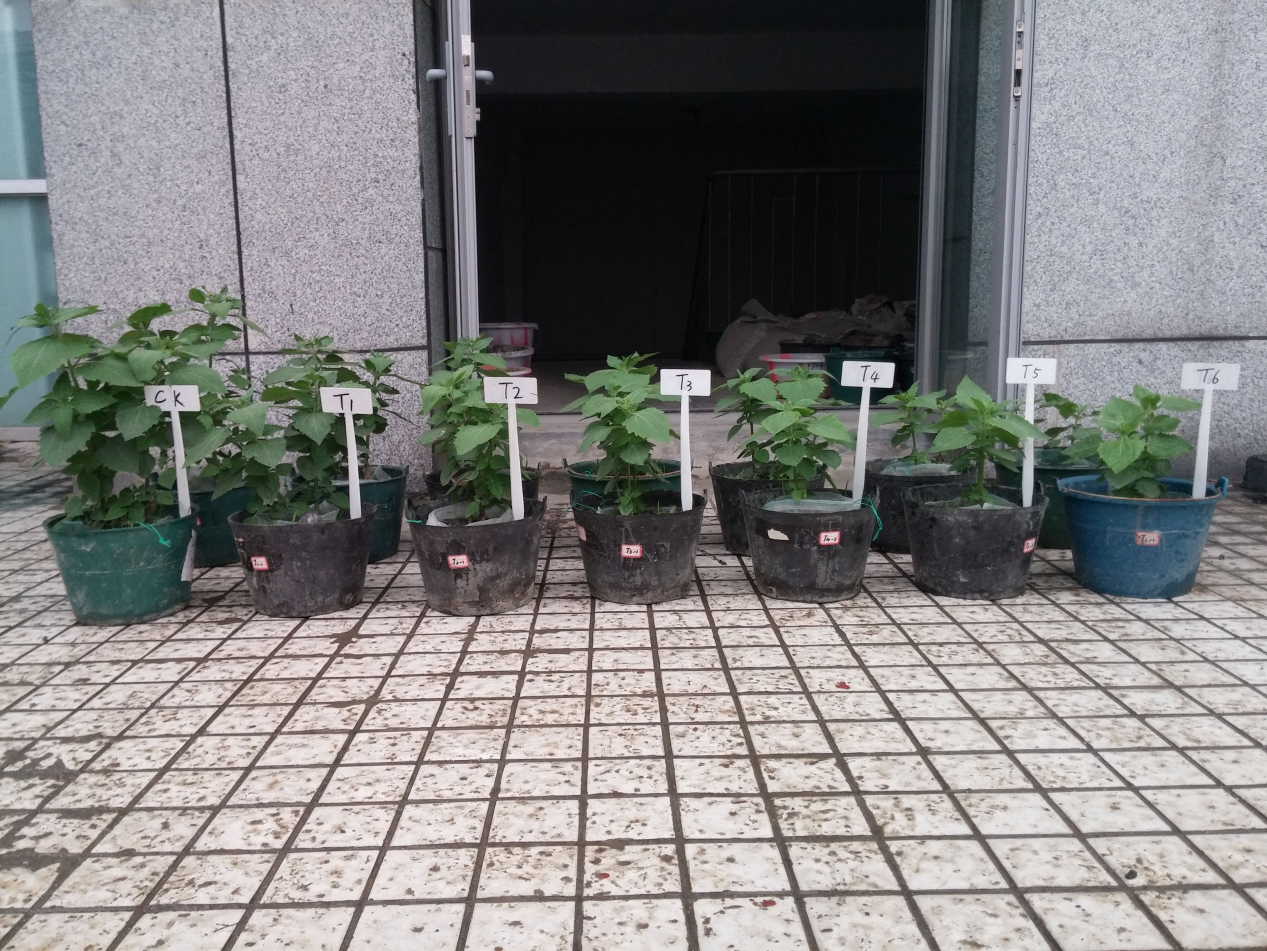
The growth of *S. orientalis* in potted plants under Cd stress.

The experiment was carried out in the greenhouse of Sichuan Agricultural University, Chengdu (103°52′E, 30°43′N), with an average air temperature of 27°C during the day and 18°C at night, and humidity of 75-80%. The physicochemical properties of soil were shown in **Table 1**. Stones and plant residues were removed after air-dried grinding and passed through a nylon sieve with a particle size of 4 mm. Each pot (25 cm×20 cm) was filled with 6.0 kg of soil and 4.2 g of compound fertilizer (N:P2O:K2O=17:17:17) prepared before and was mixed with Cd in solution (prepared by dissolving analytical grade CdCl2·2.5H2O) at 0 (control), 5, 10, 25, 50, 100, and 150 mg kg-1, respectively (**Figure 1**). Cd was added to the soil in the form of CdCl2·2.5H2O solution at one time, and the soil was stirred every day to ensure that the concentration and fraction of Cd reached a balanced state. After thoroughly mixing the soil with CdCl2·2.5H2O, the soil sample was stabilized for 40 days and then used for all subsequent experiments. 300-mesh nylon bags about 15 cm in diameter were selected as root bags and filled with prepared Cd-contaminated soil inside and outside. Three plants were cultivated in each pot, and three pots were set in each treatment. Soil moisture maintained at 80% by timely replenishing water during the plant cultivation.

**Table 1:** The physicochemical properties of soil.

Physicochemical properties of soil	Content
Clay	25.3%
Silt	40.1%
sand	34.6%
pH	6.35
available P	20.27 g/kg
total N	103 mg/kg
available P	15.36 mg/kg
available K	148.60 mg/kg
Cd	0.21 mg/kg

After 60 days, plants and soils were carefully harvested from pots. The rhizosphere soil and the non-rhizosphere soil were separated with nylon bags. A non-rhizosphere soil is composed of five samples from 2 cm away from the root bag, the remained soil adhered to the root hairs was collected as the rhizosphere soil (Su et al., 2009).

### 2.2 Soil property analysis

To evaluate the status of the soil environment, several target indices such as soil pH, soluble dissolved organic carbon (DOC), water-soluble organic carbon (WSOC), and readily oxidizable carbon (ROC), the collected soil was sieved through a 2 mm sieve to ensure homogeneity. The pH of rhizosphere and bulk soils was determined by a glass electrode with an l:2.5 soils: water ratio. DOC, WSOC and ROC in soil were extracted with 50 mL1 mol L^-1^ KCl, deionized water and 200 mmol L^-1^ KMnO4 solution in turn, and measured with total organic carbon instrument (TOC-VCPH, Shimadu, Japan) following the study of Li et al. (2016).

### 2.3 Fraction and determination of Cd

There are 4 different forms of Cd in soils that were analyzed in this study. The method of improved European Community Bureau of Reference (BCR) sequential extraction was used to determine the metal partitioning)(Liu et al., 2020). The first is to extract the acid-soluble Cd: 40 mL 0.11 M CH_3_COOH (16 h, room temperature). The second step is to extract the reducible Cd: 40 mL 0.5 M NH_2_OH·HCl (16 h, room temperature). The third step is to extract the oxidizable Cd: 10 mL 8.8 M H_2_O_2_ + 10 mL 8.8 M H_2_O_2_ + 50 mL 1 M NH_4_OAc (1 h, 85 C; 1 h, 85 C; 16 h, room temperature). After each extraction, the supernatant was taken for determination, and the residue was cleaned with deionized water before the next step. Residual: digested by 10 mL HF + 10 mL HNO_3_ + 3 mL HClO_4_, and dissolve with 1 mL solution (HNO_3_: H_2_O =1: 1. The Cd concentrations in all the extracts were analyzed with AAS (MKII, M6, Thermo Electron Corporation, USA).

### 2.4 Soil enzyme and bacterial analysis

Soil urease, phosphatase, and catalase activities were determined by conventional methods (Ma et al., 2015). Referring to (Vance et al., 1987), soil microbial biomass carbon (MBC) was analyzed by the method of chloroform fumigation- K_2_SO_4_ extraction. Basal respiration was determined by following (Wardle et al., 1993). The metabolic quotient of soil microorganisms was calculated by the ratio of soil basal respiration intensity to MBC. Soil ammonification activities were expressed as mg ${\text{NH}}_{4}^{+}$ -N per kg dry soil per day, and the content of ${\text{NH}}_{4}^{+}$ -N was determined by distillation and Kjeldahl method. Soil nitrification were expressed as the percent reduction after inoculation. The specific test steps refer to the method of Zhang et al. (2021).

### 2.5 Data and statistical analysis

The data were analyzed by one-way analysis of variation (ANOVA). T test was used to compare the significant difference between rhizosphere and non-rhizosphere in the same treatment, and the multiple comparison between treatments was performed by Duncan’s new multiple range methods (*P* < 0.05). Correlation regression analysis was used for some indexes. Redundancy analysis was used to examine the relationships between soil enzyme activity, soil microorganism quantity, and main soil properties using Canoco 4.5. Some statistical graphs were drawn by Origin 9.0.

## 3 Result

### 3.1 Concentration of Cd fractions in rhizosphere soil

**Figure 2:**
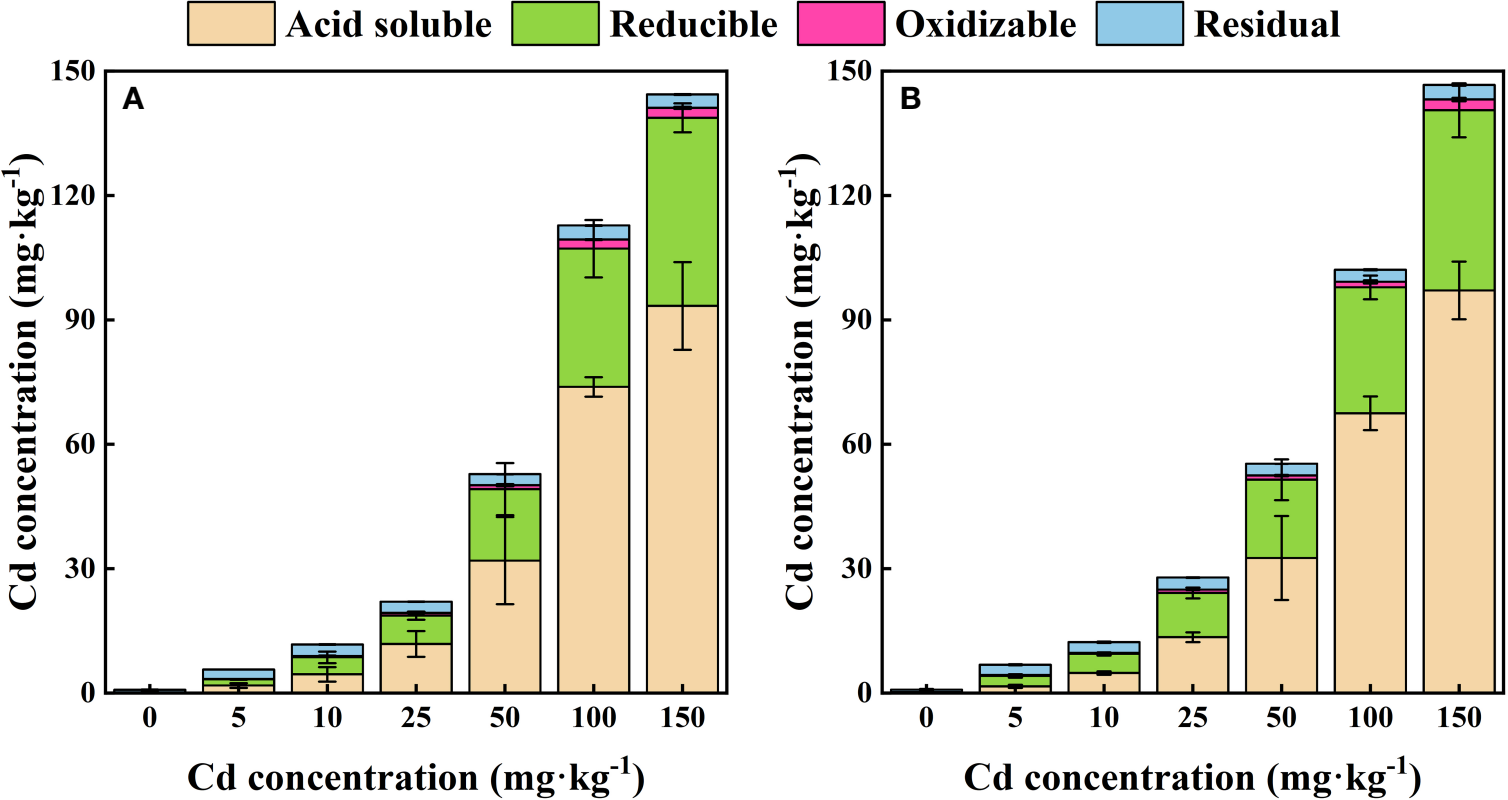
Proportion of Cd speciation in the rhizosphere **(A)** and bulk **(B)** soil of *S. orientalis*.

Increased concentrations of all four different Cd forms in rhizosphere and bulk soil can be found when higher Cd concentration were added (**Figure 2**). With the increase of Cd application, the proportion of acid-soluble Cd in rhizosphere soil obviously increased (*P* < 0.05) from 31.87% to 64.60%, while the value in bulk soil changed from 23.53% to 66.30%. For reducible Cd in rhizosphere soil, it accounted for 26.00 - 34.49 % in rhizosphere soil and 29.55%-38.29% in bulk soil when the added Cd concentration increased from 0 to 150 mg kg^-1^. There was no significant difference in the proportion of oxidizable Cd among all Cd treatments (*P* > 0.05) in rhizosphere and bulk soil. The proportion of residual Cd in total Cd significantly decreased (*P* < 0.05) from 40.36% to 2.25% for rhizosphere soil, and from 36.13% to 2.39% for bulk soil.

### 3.2 Variations of soil properties

**Figure 3:**
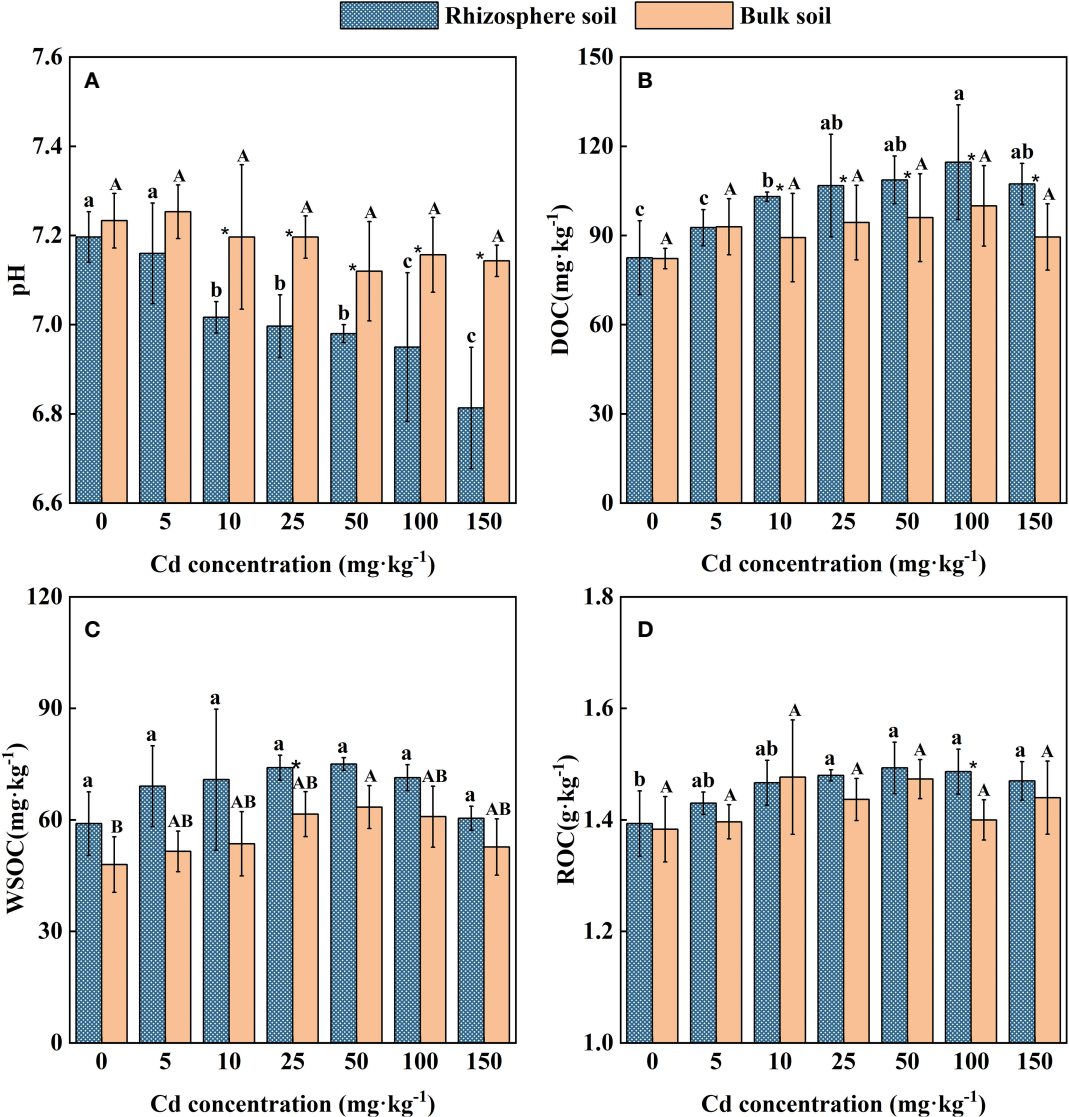
Effects of Cd on pH **(A)**, DOC **(B)**, WSOC **(C)**, ROC **(D)** in the rhizosphere and bulk of *S. orientalis*. Error bar represents standard deviation (n=3). * indicate significant difference at P < 0.05 level in the same treatment between rhizosphere and bulk soil, different lower case letter (upper case letter) at the column indicate significant difference at P < 0.05 level in the rhizosphere soil (in the bulk soil) according to ANOVA and Duncan tests, the same as follows.

The variation trend of pH in rhizosphere and bulk soil of *S. orientalis* has an obvious difference (**Figure 3A**). The Cd stress did not change the pH in the bulk soil of all treatments, while it led to lower pH (0.2 units on average) in the rhizosphere soil of *S. orientalis*. The pH value of rhizosphere soil within the range of 10 – 150 mg kg^-1^ Cd use concentration was significantly decreased than those values at the Cd use concentration of 0 mg kg^-1^ and the bulk soils (*P* < 0.05). It can be found that the pH value of rhizosphere soil gradually decreased with the increase of added Cd concentration.

As shown in **Figure 2B**, a low concentration of external Cd (5 mg kg^-1^) had no significant effect on DOC content in rhizosphere soil (*P* > 0.05), while the continuous increase of Cd supply (Cd ≥ 10 mg kg^-1^) significantly increased the DOC content of rhizosphere soil (*P* < 0.05). The increase of external Cd concentration had no significant influence on DOC content in bulk soil (*P* > 0.05). Under 100 mg kg^-1^ Cd treatment, DOC content in rhizosphere soil reached the maximum value of 114.66 mg kg^-1^, which was 28.39% higher than that of the control.

Similarly, the increase of external Cd concentration had no significant impact on bulk soil ROC content (*P* > 0.05). A low concentration of Cd (5-10 mg kg^-1^) had no significant effect on the ROC of rhizosphere soil (*P* > 0.05). When Cd concentration is larger than 25 mg kg^-1^, higher ROC content in rhizosphere soil can be seen from **Figure 3** (*P* < 0.05). At the Cd use concentration of 50 mg kg^-1^, ROC content in rhizosphere soil reached the maximum value of 1.49 g kg^-1^, which was 6.98% higher than that of the control.

Different from changes in soil DOC and ROC, Cd stress had little effect on WSOC in both rhizosphere and bulk soil. Except for the Cd use concentration of 25 mg kg^-1^ and 50 mg kg^-1^, there was no significant difference in WSOC concentration between rhizosphere and bulk soil in all treatments (**Figure 2C**). Meanwhile, the WSOC content of rhizosphere soil maintained small difference in these Cd use concentrations (*P* > 0.05).

### 3.3 Soil microorganism

**Figure 4:**
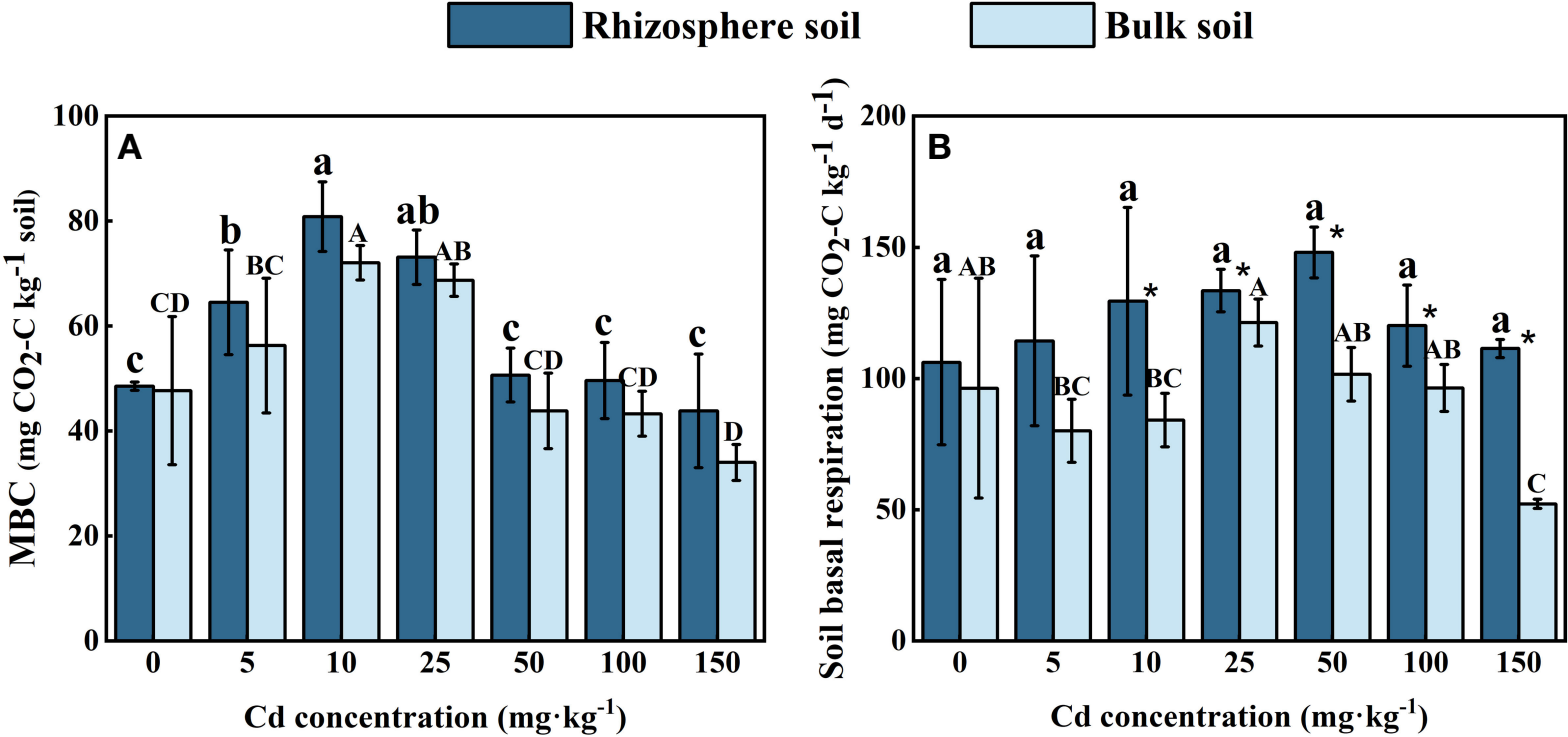
Effects of Cd on MBC **(A)** and soil basal respiration **(B)** in the rhizosphere soil and bulk soil of *S. orientalis*. Error bar represents standard deviation (n=3). * indicate significant difference at P < 0.05 level in the same treatment between rhizosphere and bulk soil, different lower case letter (upper case letter) at the column indicate significant difference at P < 0.05 level in the rhizosphere soil (in the bulk soil) according to ANOVA and Duncan tests, the same as follows.

As shown in **Figure 4A**, the concentration of MBC in both the rhizosphere and bulk soil of *S. orientalis* increased at first and then decreased with the Cd use concentration raised. The maximum concentrations of MBC in rhizosphere soil (80.88 mg kg^-1^) and in bulk soil (72.11 mg kg^-1^) both occurred at the Cd use concentration of 10 mg kg^-1^, and increased by 50.56% and 51.11% compared with the control. When the Cd use concentration reached 150 mg kg^-1^, the concentration of MBC in rhizosphere and bulk soil both dropped down to the lowest value. And the MBC concentration in rhizosphere soil decreased by only 9.74% compared with the control, while in bulk soil was significantly lower than the control (*P < 0.05*), decreased by 28.67%.

The BR intensity of all rhizosphere soil was higher than that of bulk soil (**Figure 4B**). In comparison to the control, Cd stress did not significantly inhibit BR in the rhizosphere of *S. orientalis* (*P* < 0.05), and each treatment results increased by 7.65%-39.35%. More complex variation occurred in bulk soil. The maximum value of bulk soil basal respiration intensity occurred at the Cd use concentration of 25 mg kg^-1^, which increased by 25.0% compared with the control treatment. And then the BR intensity in bulk soil decreased gradually with the increase of Cd added content. The minimum value was decreased by 45.79% compared with the control.

**Figure 5:**
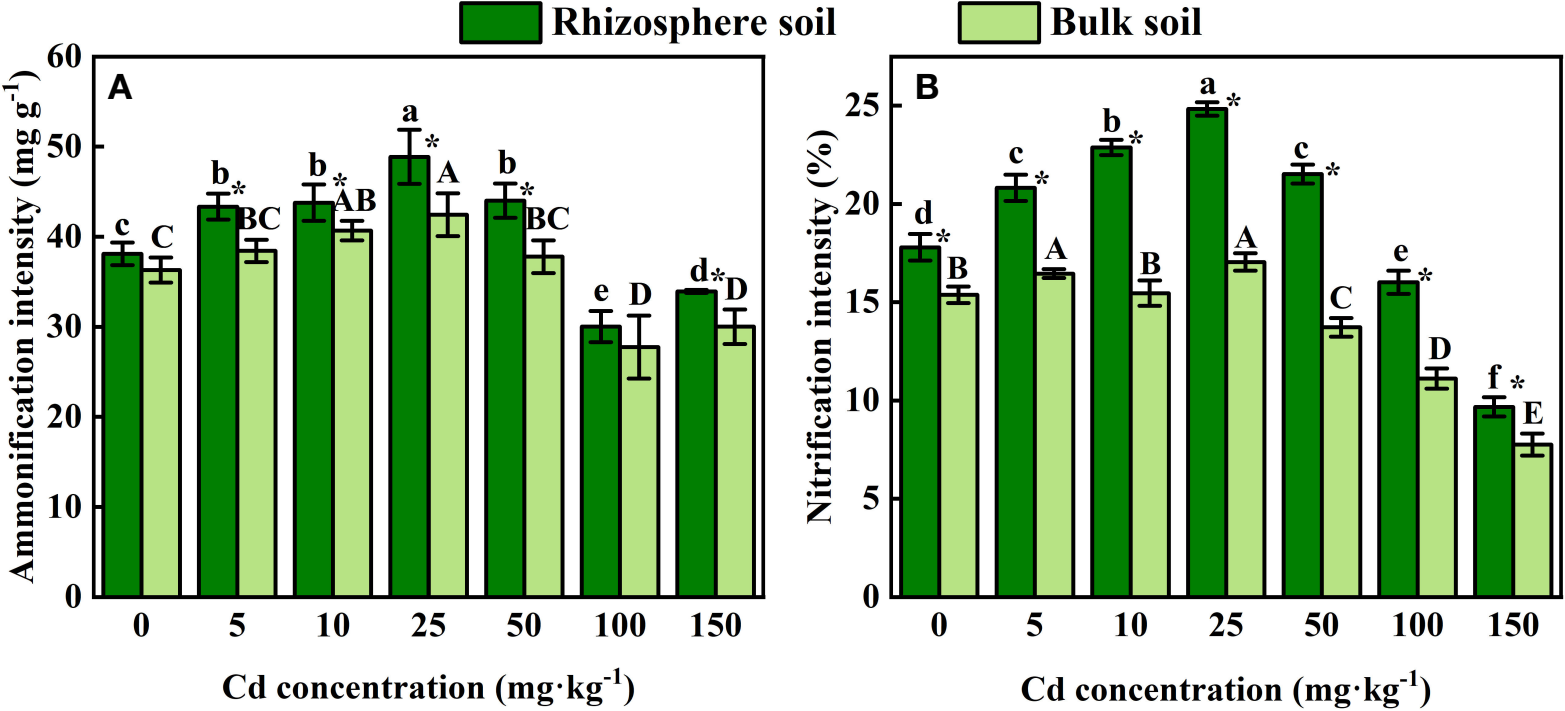
Error bar represents standard deviation (n=3). * indicate significant difference at P < 0.05 level in the same treatment between rhizosphere and bulk soil, different lower case letter (upper case letter) at the column indicate significant difference at P < 0.05 level in the rhizosphere soil (in the bulk soil) according to ANOVA and Duncan tests, the same as follows.

As shown in **Figure 5**, the intensity of ammonification and nitrification in both rhizosphere soil and bulk soil of *S. orientalis* significantly increased within the range of 5 – 50 mg kg^-1^ Cd concentration. The maximum intensity of both ammonification and nitrification was achieved at Cd use concentration of 25 mg kg^-1^. With the continued increase of Cd use concentration from 50 mg kg^-1^, both of them showed a decreasing trend in rhizosphere and bulk soil. The intensity of nitrification and ammonification in rhizosphere soil was significantly higher than that in bulk soil.

### 3.4 Soil enzyme activity

Urease activity in rhizosphere and bulk soil increased at first at low concentrations of external Cd treatment (0-10 mg kg^-1^), subsequently reduced at high concentrations of Cd treatment (50-150 mg kg^-1^). At Cd use concentration of 5 mg kg^-1^, no significant effect on urease activity can be found. However, with the increase of external Cd concentration (10-100 mg kg^-1^), the activity of urease in rhizosphere soils was significantly higher than that in bulk soils (*P* < 0.05). The maximum rhizosphere soil urease activity of 0.40 mg ammonia g^-1^ d^-1^ was achieved at the Cd use concentration of 10 mg kg^-1^, and the value were increased by 13.21% compared with the control.

**Figure 6:**
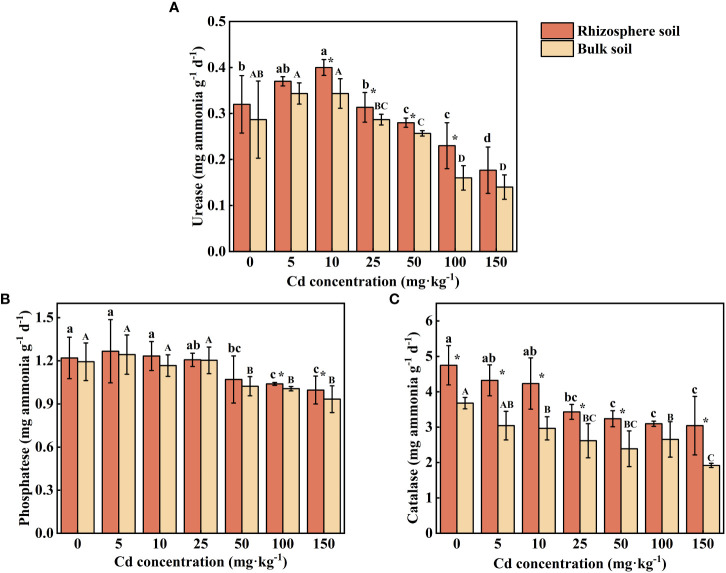
Effects of Cd on activity of urease **(A)**, phosphatase **(B)** and catalase **(C)** in the rhizosphere soil and bulk soil of *S. orientalis*. Error bar represents standard deviation (n=3). * indicate significant difference at P < 0.05 level in the same treatment between rhizosphere and bulk soil, different lower case letter (upper case letter) at the column indicate significant difference at P < 0.05 level in the rhizosphere soil (in the bulk soil) according to ANOVA and Duncan tests, the same as follows.

The changing trend of phosphatase was the same as that of urease. Phosphatase activity in rhizosphere soil was significantly higher than that of bulk soil under the treatment of the two highest concentrations of Cd (*P* < 0.05), while there was no significant difference in phosphatase activity between rhizosphere and bulk soils treated with low and medium concentrations of Cd (5-50 mg kg^-1^) (**Figure 6B**, *P* > 0.05). The maximum phosphatase activity in rhizosphere soil (1.27 mg phenol g^-1^ d^-1^) and in bulk soil (1.24 mg phenol g^-1^ d^-1^) occurred at the Cd use concentration of 5 mg kg^-1^. As the supply of Cd increased, the phosphatase activity decreased significantly (*P* < 0.05) 9.56%-17.76% in rhizosphere soil and 14.25%-21.79% in bulk soil compared with the control.

The catalase activity increased in both rhizosphere and bulk soil with increasing Cd use concentration in all samplings (**Figure 6C**). The activity of catalase in rhizosphere soil was significantly higher than that in bulk soil except for one condition (100 mg Cd kg^-1^, *P* < 0.05). Compared with the control, low Cd use concentrations (5-10 mg kg^-1^) did not significantly inhibit the catalase activity of rhizosphere soil (*P* > 0.05), while high and medium Cd use concentrations (≥25 mg kg^-1^) can decrease the catalase activity, which is decreased by 27.72%-35.93%.

## 4 Discussion

### 4.1 Rhizosphere acidification and organic carbon accumulation drives Cd activation

Cd activation refers to the process by which Cd in soil changes from stable fractions (acid-soluble and reducible Cd) to labile fractions (oxidizable and residual Cd), and the bioavailability of Cd increases during this process. We followed the objective to measure the related indexes and Cd morphological changes of soil. Relevant hypothesis was confirmed by the change of soil physical and chemical properties caused by rhizosphere effect. These changes are important to activate HMs. BCR extraction procedures were used to evaluate Cd availability. According to the BCR sequential extraction method, HMs in soils can be classified into acid-soluble, reducible, oxidizable, and residual fractions, among which the acid-soluble fraction has the highest bioavailable and the residual fraction has the lowest bioavailability (Anju and Banerjee, 2010; Yang et al., 2017; Huang et al., 2020). In general, the mobility of HMs in the soil is relatively weak, and the fractions with high bioavailability, such as acid-soluble fraction, are not high (Li et al., 2011). *S. orientalis* can absorb large amounts of Cd from the soil, so there must be an activation process to change the morphology and bioavailability of Cd. As can be seen from **Figure 2**, with the increase of the concentration of Cd, the proportion of weak-acid extracted Cd from the rhizosphere increased gradually, the proportion of reducible and oxidizable Cd changed little, while the proportion of residue Cd with the lowest bioavailability decreased significantly. This is consistent with the research results of *R. globose* under Cd treatment (Wei and Twardowska, 2013). The concentration of acid-soluble fraction in rhizosphere soil was lower than that in bulk soil under all treatments (**Figure 2**) because Cd absorption rate was higher than the activation rate. Some reports hold that the mobility and bioavailability of HMs in soil are mainly influenced by physicochemical properties such as soil pH and organic matter content (Bravin et al., 2012; Seshadri et al., 2015; Huang et al., 2020).

**Figure 7:**
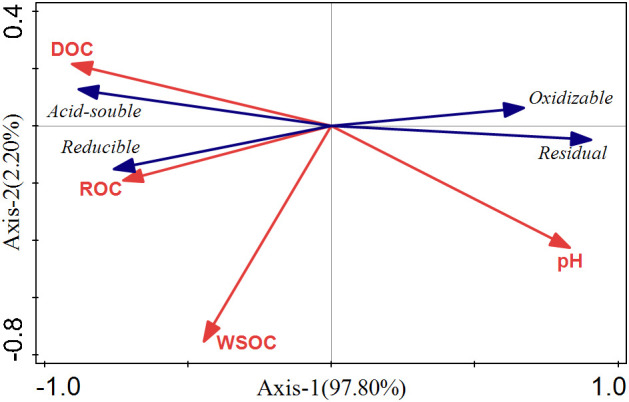
RDA ranking of Cd form and soil properties in rhizosphere soils.

Rhizosphere soil pH can significantly impact the bioavailability and toxicity of HMs in soil, which could result in the desorption of HMs from soil particles by reducing the pH (Niu et al., 2021; Ning et al., 2022). These effects will further promote the uptake of HMs by plants (Li et al., 2014; Mimmo et al., 2014). In this study, external Cd induced a reduction of pH in the rhizosphere (**Figure 3A**), as Zhan et al. (2018) observed in *A. wardii*. Redundancy analysis (RDA) also showed that the pH of rhizosphere is closely related to Cd fractions, and rhizosphere acidification could increase Cd concentrations in four forms (**Figure 7**). Rhizosphere acidification can be attributed to the secretion of organics including organic acid and carbohydrates by hyperaccumulator (Kushwaha et al., 2015; Niu et al., 2020). Rhizosphere acidification is a soil chemical process occurring in the root-soil interface (Antoniadis et al., 2017). Consequently, acidification of the rhizosphere leads to the transformation of Cd from low bioavailable (reducible) to high bioavailable (acid-soluble) (**Figures 2A**).

In addition to rhizosphere acidification, the concentration of active organic carbon components can play important role in activation of HMs in soil solution (Clemente and Bernal, 2006; Kim et al., 2010). We divided the activated carbon components into DOC, ROC, and WSOC. These organic compounds are derived from the mineralization of soil organic matter and root exudation (Pii et al., 2015). DOC can be used as a carrier for accelerating hyperaccumulator uptake of HMs (Huang et al., 2020). Many studies have confirmed the activation effect of DOC on HMs (Wenzel et al., 2003; Li et al., 2011; Li Z, et al., 2021). In this study, DOC induced an increase in high bioavailability Cd fractions (acid-soluble and reducible Cd) (**Figure 7**). DOC can form soluble organometallics complexes with HMs, or substitute HMs for preferentially adsorbed on the soil surface, to reduce the adsorption of HMs on the soil surface and improve its bioavailability (Cornu et al., 2011; Bravin et al., 2012; Welikala et al., 2018). Therefore, DOC was one of the important factors promoting the activation and hyperaccumulation of Cd in *S. orientalis* under Cd stress.

Compared to DOC, ROC accounted for a higher proportion of labile soil organic carbon components and had a faster turnover, so it was more sensitive to soil environmental changes (Zhang et al., 2020). ROC is organic carbon that is easily oxidized by potassium permanganate, and its content is easily affected by anthropogenic activities (Jiang M, et al., 2022). In our study, the supply of exogenous Cd could increase the content of ROC in rhizosphere soil, especially at medium and high concentrations (Cd ≥ 25 mg kg^-1^) significantly increased (P < 0.05) (**Figure 2D**). Combined with the results that ROC content in the bulk soil had no significant effect (P > 0.05), we can conclude that the ROC variation is caused by the root system. The elevated level of ROC may be due to Cd toxicity caused by a declining trend of soil pH with the increasing of Cd stress increases (**Figure 2A**), which may damage the roots and increase root exudation (Li et al., 2015). This has been confirmed in previous studies (Hurisso et al., 2014). At present, few studies have focused on the relationship between the change of ROC content in rhizosphere soil and the activation of HMs. However, our RDA showed that the content of ROC is closely related to the Cd availability (**Figure 7**). ROC is a large order of magnitude of active organic carbon components, mainly originate from the mineralization of microbial and plant root exudates (De Conti et al., 2018; Li G, et al., 2021; Jiang O, et al., 2022). Concerning the latter, root exudates included amino acids, low molecular weight organic acids, and soluble sugars, and their release was beneficial to the accumulation of ROC (Xiao et al., 2018; Liu et al., 2020; Li G, et al., 2021). From the composition of ROC, these substances play an important role in improving the mobility and the absorption of HMs by plants (Kim et al., 2010; Madej, 2016; Jiang M, et al., 2022). Consequently, the increase of rhizosphere secretions of *S. orientalis* under Cd stress promotes the accumulation of ROC, thus increasing the bio-availability of Cd and facilitating the hyperaccumulation of Cd by *S. orientalis*. However, some limitations are worth noting. Although our hypotheses were supported statistically, our samples did not analyze the specific composition of root exudates. Future work should include the effect of specific root exudate composition on Cd morphology.

WSOC is the most mobile and susceptible fraction of soil organic carbon (Li et al., 2015). Previous studies showed that WSOC content was not significantly affected by plant planting systems and soil depth, but was mainly controlled by precipitation leaching (Sharma et al., 2014; Guo et al., 2018). In this study, the content of WSOC was not significantly affected by Cd stress (**Figure 3C**), and there was no significant correlation between WSOC and the form of Cd in rhizosphere soil (**Figure 7**; **Table 2**). It can be inferred that WSOC played a limited role in Cd activation.

**Table 2:** Correlations between partial soil properties and Cd speciation in the rhizosphere and non-rhizosphere of *S. orientalis* across all samples.

	Soil properties	Acid soluble	Reducible	Oxidizable	Residual
Rhizosphere soil	pH	-0.695**	-0.567**	0.502*	0.705**
	DOC	0.741**	0.718**	-0.644**	-0.780**
	WSOC	0.299	0.427	-0.393	-0.349
	ROC	0.280	0.449*	-0.353	-0.351
Bulk soil	pH	-0.409	-0.102	0.184	0.411
	DOC	0.261	0.316	-0.290	-0.314
	WSOC	0.470*	0.402	-0.505*	-0.483*
	ROC	0.304	0.317	-0.270	-0.356

Values are Pearson correlation coefficients, *indicated the correlations were significant at P < 0.05, **indicated the correlations were significant at P < 0.01.

### 4.2 The increase of microorganism and enzyme activity is the mechanism of *S. orientalis* tolerance to Cd

In addition to accumulating high concentrations of HMs, hyperaccumulators also have the mechanism of tolerating and sustaining highly toxic HMs (Clemens, 2017; Pasricha et al., 2021). Hypertolerance is the key property that allows plants to avoid HM poisoning (Chaney et al., 1997). The properties can be divided into *in vivo* and *in vitro* according to the site of action. In vivo, chelation/sequestration deal with the toxicity of accumulated metal ions, while the anti-oxidative defense system of plants is used to cope with HM-induced oxidative damage (Manara, 2012; Macnair et al., 2000; Yan et al., 2020; Feng et al., 2021). These indicators have been thoroughly studied in previous researches (Lux et al., 2011; Pasricha et al., 2021; Yaashikaa et al., 2022), but the role of rhizosphere in plant tolerance is often ignored.

**Figure 8:**
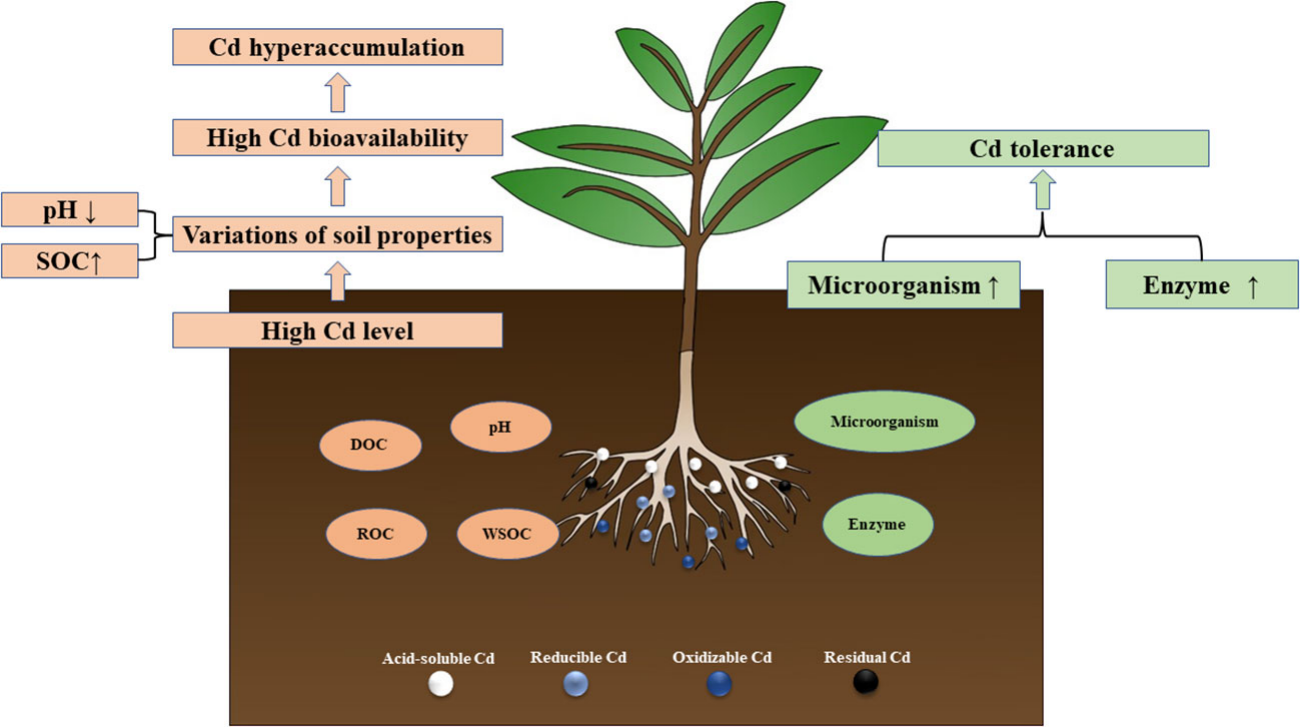
Schematic of the mechanism of the activation and tolerance of *S. orientalis* Cd.

*In vitro*, soil enzyme activity is an important index reflecting soil quality and vitality (Zhang et al., 2015a). Their activity is related to the number of soil microorganisms, soil conditions, and plant growth (Jaiswal and Pandey, 2018; Gao et al., 2020). Therefore, soil enzyme activity can accurately reflect the response of soil microorganisms to HMs exposure during phytoremediation (Cao et al., 2020). In our study, with the increase of Cd supply, the enzyme activities showed a decreasing trend (**Figure 6**). Cd can reduce enzyme activity by denaturing the enzyme protein, binding with the enzyme substrate complex, and inhibiting microbial activity (Ma et al., 2015; Cao et al., 2020). However, medium and low concentrations of Cd (Cd ≤ 10 mg kg^-1^), *S. orientalis* rhizosphere maintained the activity of rhizosphere soil enzymes so that they are not significantly inhibited. The role of soil microorganisms in detoxifying pollutants, facilitating nutrient cycling, and producing essential compounds for both microorganisms and plants has been demonstrated (Ma et al., 2015). Urease catalyzed hydrolysis of urea is one of the important sources of plant nitrogen, catalase can effectively remove the hydrogen peroxide toxicity caused by stress environment, and phosphatase can help maintain a good supply of phosphorus in Cd-contaminated soil (Weng et al., 2014; Ma et al., 2015; Sturikova et al., 2018; Wang et al., 2018). This suggests that rhizosphere plays an important role in maintaining nutrient supply and detoxification of plants in Cd-contaminated soils through soil enzymes. To sum up, the rhizosphere environment had a complex effect on soil enzyme activities, and the enhancement of plant rhizosphere soil enzyme activities improved the conversion of nitrogen, phosphorus, and organic matter in Cd contaminated soil, maintained soil fertility, and was conducive to promoting the growth of *S. orientalis*, and also promoted the absorption of Cd by plant roots to a certain extent (**Figure 8**).

Microorganisms in rhizosphere soils have been proven to detoxify of HMs, and promote nutrient cycling and transfer of soil energy (Guo et al., 2019). Therefore, rhizosphere microorganisms play an important role in improving the efficiency of phytoremediation (Cao et al., 2020). MBC is an active carbon reservoir in the soil micro-ecosystem, which can be a sensitive indicator of soil microbial biomass and soil quality, its enhancement of MBC may help increase crop productivity and sustainability (Liu et al., 2011; Zhan et al., 2018). Soil basal respiration was accepted as a sensitive indicator of environmental stresses on soil microbial community microbial stress, and microorganisms can decompose soil organic carbon and produce energy through soil basal respiration (Bian et al., 2015; Zhou et al., 2017). These indicators are often used to characterize environmental and microbial changes. In general, high concentrations of HM pollution can negatively affect the number and activity of microorganisms in the soil (Pan and Yu, 2011). Our results showed that both MBC content and soil basal respiration in rhizosphere soil increased in all Cd treatments compared with the control these in the control treatment (**Figure 4**). At the same time, the rhizosphere soils were always higher than bulk soils. This may be ascribed to the exudates of *S. orientalis* rhizosphere under Cd stress promoting the improvement of soil enzyme activities and was beneficial to the nutrient cycling in the soil and the survival of microorganisms (Li Z, et al., 2021). However, as the concentration increased, the bioavailability and mobility of Cd also increased (Zhan et al., 2018). This phenomenon leads to a gradual increase in the inhibition of microbial activity. Similar results have been found in other studies (Bérard et al., 2016; Jaiswal and Pandey, 2018). These results confirm that a series of rhizosphere activities maintain the microbial activity of rhizosphere soil and promote Cd tolerance to *S. orientalis*.

Ammonification and nitrification are two basic processes of N cycling in soil, which convert N into two main nitrogen forms, ammonium ( ${\text{NH}}_{4}^{+}$ ) and nitrate ( ${\text{NO}}_{3}^{-}$ ), that are efficiently taken up by plant roots (Salsac et al., 1987; Beeckman et al., 2018). Moreover, root growth is stimulated by ammonium and nitrate that provide sufficient nutrients (O’Brien et al., 2016; Xuan et al., 2017). Ammonification and nitrification of soil microorganisms are also important factors to maintain plant growth and alleviate heavy metal toxicity in plants(Li et al., 2019; Wang et al., 2021). Our results showed that ammonification and nitrification were inhibited when Cd concentration is greater than 100 mg kg^-1^, whereas enhanced at Cd concentration of 5-50 mg kg^-1^. And they were significantly higher in rhizosphere soil than these in bulk soil (**Figure 5**). The intensities of ammonification and nitrification were mainly influenced by the microhabitats. In addition, high concentrations of Cd will lead to microbial inactivation affecting the intensity (Wang et al., 2021). The rhizosphere exudates improved the microbial activity and alleviated the stress of Cd to *S. orientalis* when the Cd supply was low. These results indicate that ammonification and nitrification of rhizosphere soil play an important role in promoting tolerance of *S. orientalis*.

## 5 Conclusion

Cd activation refers to the process by which Cd in soil changes from stable fractions (acid-soluble and reducible Cd) to labile fractions (oxidizable and residual Cd), and the bioavailability of Cd increases during this process. Meanwhile, compared with the treatment without Cd, exogenous application of Cd resulted in rhizosphere acidification and accumulation of organic carbon, which induces Cd activation. In turn, accumulation of active organic carbon alleviated the inhibition effects on microbial and enzymatic activities in rhizosphere soil. These improvements presented great benefit for *S. orientali* tolerance in Cd-contaminated soils. Our findings provide new insights into the hyperaccumulation and detoxification of HMs by hyperaccumulator root-soil systems and provide possibilities for improving the phytoremediation efficiency of contaminated soils.

## Data availability statement

The original contributions presented in the study are included in the article/supplementary material. Further inquiries can be directed to the corresponding author.

## Author contributions

JYX: data curation; investigation; writing-original draft. XX: funding acquisition; supervision; writing-review and editing. SZ: project administration; writing-review and editing. ZY: software; writing-review and editing. GW: resources; writing-review and editing. TL: resources; writing-review and editing. YP: validation; writing-review and editing. WZ: validation; writing-review and editing. CX: project administration; writing-review and editing. GL: resources; writing-review and editing. ZC: visualization; writing-review and editing. JRX: software; writing-review and editing. ZP: validation; writing-review and editing. All authors contributed to the article and approved the submitted version.
